# Examining the Roles of Problematic Internet Use and Emotional Regulation Self-Efficacy on the Relationship Between Digital Game Addiction and Motivation Among Turkish Adolescents

**DOI:** 10.3390/bs15030241

**Published:** 2025-02-20

**Authors:** Öner Çelikkaleli, Rıdvan Ata, Muhammet Mustafa Alpaslan, Zafer Tangülü, Özgür Ulubey

**Affiliations:** 1 Department of Psychological Counseling and Guidance, Mugla Sitki Kocman University, Muğla 48000, Türkiye; onerckaleli@mu.edu.tr; 2 Department of Instructional Technologies, Mugla Sitki Kocman University, Muğla 48000, Türkiye; 3 Department of Mathematics and Science Education, Mugla Sitki Kocman University, Muğla 48000, Türkiye; mustafaalpaslan@mu.edu.tr; 4 Department of Social Studies Education, Mugla Sitki Kocman University, Muğla 48000, Türkiye; zafertangulu@mu.edu.tr; 5 Department of Curriculum and Instruction, Mugla Sitki Kocman University, Muğla 48000, Türkiye; oulubey@mu.edu.tr

**Keywords:** academic motivation, digital game addiction, emotional regulation self-efficacy, problematic internet use, Turkish adolescents

## Abstract

Digital game addiction and problematic internet use have emerged as significant issues, attracting growing attention from educators, psychologists, and policymakers. This study aimed to examine the mediating role of emotional regulation self-efficacy and the moderating role of problematic internet use in the effect of digital game addiction on academic motivation in Turkish adolescents. A correlational research method was utilized to address research questions. A total of 1156 high school students voluntarily participated in the study. Self-report questionnaires (the Short Academic Motivation Scale, Digital Game Addiction Scale, Regulatory Emotional Self-Efficacy Scale and Young’s Internet Addiction Scale Short Form) were used to collect data in 2024. In the analysis of the data, Pearson Product Moment Correlation Coefficient, mediator and moderator analyses were conducted using statistical software. The analysis provided evidence of the negative effect of digital game addiction on academic motivation. Additionally, emotional regulation self-efficacy was found to partly mediate the relationship between digital game addiction and academic motivation. Furthermore, problematic internet use moderated the relationship between digital game addiction and academic motivation in adolescents. The results suggested enhancing adolescents’ emotional regulation self-efficacy and reducing problematic internet use are crucial steps towards mitigating the negative effects of digital game addiction on academic motivation.

## 1. Introduction

Along with technological development, digital tools including the internet and digital gaming have become integral parts of individuals’ lives. High school-aged students, in particular, have increasingly engaged in online activities, dedicating significant amounts of time to the internet and the digital realm (Nogueira-López et al., 2023). This engagement influences various aspects of their lives, including the fulfillment of basic psychological needs, identity formation and academic development (A. Chen, 2019; Partala, 2011; Raiziene et al., 2022). Moreover, during the COVID-19 pandemic, adolescents were banned from non-essential outdoor activities which led them to be more exposed to digital platforms and the internet (Islam et al., 2023). This increased online presence raises concerns about the potential for risky or hazardous behaviors in the digital environment (Nogueira-López et al., 2023).

Digital game addiction is often associated with educational and health problems including amotivation, unhealthy nutritional habits, vision and hearing-related diseases, skeletal problems, sleep disorders, and depression (Örnek & Gündoğmuş, 2022). The global prevalence of digital game addiction ranges from 1.3% to 9.9% (World Health Organization [WHO], 2024). However, due to increased isolation at home, greater use of digital technologies, and reduced face-to-face communication, the rate of digital game addiction has increased more rapidly among adolescents both during and after COVID-19 (Avci et al., 2023; Meng et al., 2022). Therefore, it is critical to monitor digital game addiction in today’s youth and investigate its causes and consequences to address this behavioral and educational issue (González-Cabrera et al., 2022; Yilmaz et al., 2023; Yu et al., 2022). This study examined the effect of digital game addiction on academic motivation in adolescents, focusing on the mediating role of emotional regulation self-efficacy, which is thought to function as a protective factor, and the moderating role of problematic internet use (PIU), which was considered a risk factor that might exacerbate the effect.

### 1.1. Academic Motivation

Academic motivation refers to “the process whereby goal-directed [academic] activity is instigated and sustained” (Schunk et al., 2014, p. 5) and is a critical determinant of student success in educational settings. It encompasses both the internal drive and external influences that encourage students to engage with their studies, persist through challenges, and achieve academic goals. Understanding the factors that contribute to academic motivation is essential for educators, as it directly impacts learning outcomes, student well-being, and overall academic performance.

Various theoretical approaches have been developed to explain the motivational forces behind behavior, choices, and success. For instance, some theories, such as Expectancy–Value Theory, focus on individuals’ expectations for success and the value they place on succeeding, as a way to explain their choices and motivations (Wentzel, 2019). Another widely used theory, Self-Determination Theory (SDT), examines both external and internal motivational forces driving individuals’ behavior (Wentzel & Ramani, 2016). Many studies on technological addiction have utilized SDT to explain the connection between academic motivation and addiction (Yaghoobi et al., 2024). For this reason, SDT guided the current study.

SDT, developed by Deci and Ryan (1980, 2000), is a psychological theory of human motivation that focuses on how individuals’ needs and environments influence their motivation and behavior. According to SDT, people have three fundamental psychological needs: autonomy, competence, and relatedness (Gugliandolo et al., 2020). Autonomy refers to the need to feel in control of one’s own behavior and decisions. Competence involves feeling effective and capable in interactions with the environment. Relatedness pertains to the need to feel connected to and belonging with others. When these needs are satisfied, individuals experience intrinsic motivation, meaning they engage in activities because they find them inherently rewarding or enjoyable (Deci & Ryan, 2000). SDT also distinguishes between intrinsic motivation (doing something for its inherent satisfaction) and extrinsic motivation (doing something to achieve an external goal or reward). When these needs are not met, people may seek external rewards or engage in maladaptive behaviors (such as excessive mobile phone use) to compensate (Kwon & Yu, 2020). The balance between intrinsic and extrinsic motivation can significantly impact a student’s engagement with coursework and their long-term educational trajectory.

SDT has been widely used to explain student behavior in educational settings. For example, Karaoglan Yilmaz and Yilmaz (2024) applied SDT to explore class engagement in both motivated and unmotivated students. They found that students who lacked sufficient motivation often did not attend classes regularly, struggled to maintain focus, and showed fragmented interest and attention. These students tended to withdraw when faced with challenges rather than confront them. In contrast, highly motivated students were actively engaged in lessons, came prepared, frequently asked questions, and participated in discussions. These students were willing to put in effort, dedicate ample time to their studies, stay focused, and persist through difficulties (Howard et al., 2021). Thus, academic motivation is considered a critical determinant of educational outcomes, as successful learning requires students to voluntarily engage in the learning process.

### 1.2. Digital Game Addiction and PIU

The rapid spread of digital technology has profoundly impacted adolescents, providing opportunities for learning, social interaction, and entertainment. However, concerns have emerged over excessive online activity, including digital game addiction and problematic internet use (PIU), which have significant implications for mental health, social well-being, and academic performance. Studies show that PIU affects 4.6% to 19.1% of adolescents worldwide (Anderson et al., 2017; Mihara & Higuchi, 2017).

Digital game addiction is recognized as a behavioral addiction, driven by factors such as social reinforcement and the immersive nature of games (Škařupová & Blinka, 2016; Montag et al., 2016). It negatively impacts social interactions, mental health, and academic performance (King et al., 2020; Kuss & Griffiths, 2012). Similarly, PIU involves excessive, uncontrolled internet use, often linked to anxiety, social isolation, and sleep issues (Bányai et al., 2017). Adolescents are particularly vulnerable due to their developmental stage. The overlap between gaming and other online activities, such as social media, increases the risk of developing problematic behaviors, as seen with online multiplayer games that blend social interaction and gaming (Lopez-Fernandez et al., 2015). This convergence raises concerns about addiction in the digital age and its effects on adolescent development. Both PIU and digital game addiction negatively affect academic motivation and focus, with studies showing a link between excessive screen time and poor academic performance (Gentile et al., 2011; Paterna et al., 2024). Research consistently finds a negative correlation between digital game addiction and academic motivation (Karaoglan Yilmaz & Yilmaz, 2024; Yaghoobi et al., 2024). For instance, studies show that high levels of game addiction correlate with low intrinsic and extrinsic motivation (Atasever et al., 2023). Some argue that unmet psychological needs drive individuals toward external rewards like gaming, reducing academic motivation (Gugliandolo et al., 2020), while others suggest that addiction directly leads to disengagement from learning (Karaoglan Yilmaz & Yilmaz, 2024; Yaghoobi et al., 2024). This study examines how digital game addiction impacts academic motivation in adolescents.

### 1.3. Regulatory Emotional Self-Efficacy

Regulatory emotional self-efficacy (RESE) refers to individuals’ beliefs in their ability to manage and regulate their basic emotional states (Alessandri et al., 2023). These self-efficacy beliefs are closely tied to how effectively individuals can navigate social interactions and relationships, as they influence emotional control, communication, and overall social functioning (M. Caprara et al., 2024). Eisenberg and Spinrad (2004) define emotion regulation as the process of regulating or altering the formation, intensity, or duration of physiological processes, motivational states, behavioral and attentional processes, and internal emotional states from the onset of emotion. Higher RESE is often associated with better emotional regulation, which in turn supports more adaptive and positive social behaviors (Manfredi et al., 2024). Singh and Singh (2013) argued that difficulties in emotional regulation reduce academic motivation, which can negatively impact students’ academic success. Furthermore, Collie (2024), in the Social and Emotional Competence School Model, emphasized that emotional satisfaction plays a fundamental role in need satisfaction, as described in SDT, highlighting the importance of RESE for academic motivation.

Several studies have examined the relationship between RESE and academic motivation. Trautner and Schwinger (2020) suggested that RESE can influence self-efficacy, thereby enhancing emotional regulation. Haji Vosoogh et al. (2022) found that RESE positively, directly, and significantly affects academic motivation. Other studies have provided similar findings (Badri, 2019; Chase, 2018; Duchatelet & Donche, 2019), indicating that higher RESE is linked to increased academic motivation. Conversely, a negative relationship between emotion dysregulation and academic motivation has been observed (Singh & Singh, 2013). Additionally, Çetin (2015) demonstrated that cognitive emotion regulation strategies can improve academic motivation and performance. Namaziandost and Rezai (2024), in their study utilizing technology and artificial intelligence in second language teaching, also found that RESE positively affected academic motivation.

### 1.4. Relations Among Variables and Hypotheses

Studies examining the interplay between digital game addiction and academic motivation in adolescents have consistently reported a significant negative relationship between the two (Atadokht et al., 2014; C. G. Chen & Gu, 2019; Demir & Kutlu, 2020; Haji Anzehai, 2020; Karaoglan Yilmaz & Yilmaz, 2024; Ran, 2022; Rasheed et al., 2022; Sofia Amriza et al., 2024; Sun et al., 2023). These findings highlight the detrimental impact of digital game addiction on academic motivation; however, there is limited research on the underlying mechanisms (mediation) or variables that may either intensify or mitigate this relationship.

In previous studies exploring mediating factors in the relationship between digital game addiction and academic motivation among adolescents, variables such as participation in education (Sun et al., 2023), grit training (Haramain & Afiah, 2022), mindfulness (Fu et al., 2021), fear of missing out (FoMO) (Widyana & Purnamasari, 2020), and wisdom (Yaghoobi et al., 2024) have been identified as mediators. As for moderation, the only moderating variable explored was class level in the study by Rasheed et al. (2022).

This study seeks to advance the literature by examining the mediating role of regulatory emotional self-efficacy (RESE) in the effect of digital game addiction on academic motivation, as well as the moderating role of problematic internet use (PIU) in this relationship. Investigating these variables could provide innovative insights into the mechanisms driving the negative impact of digital game addiction on academic motivation in adolescents.

**Figure 1:**
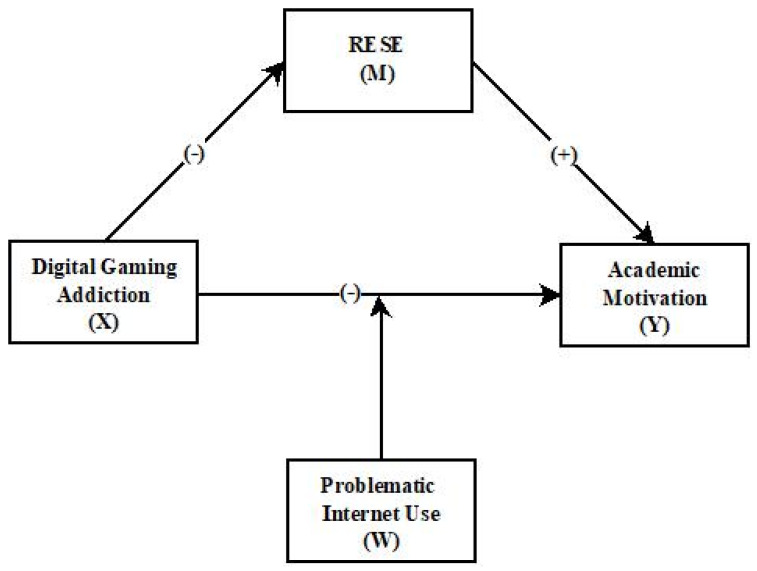
The mediating role of regulatory emotional self-efficacy in the effect of digital game addiction on academic motivation and the moderating role of PIU in adolescents.

In this context, the study aims to test the following sets of hypotheses (see Figure 1).

Hypotheses regarding the mediating role of emotion regulation:

**H1:**  *The total effect of digital game addiction (X) on academic motivation (Y) in adolescents (c) is significantly negative.*

**H2:**  *The direct effect of digital game addiction (X) on RESE (M) in adolescents (a) is significantly negative.*

**H3:**  *The direct effect of RESE (M) on academic motivation (Y) in adolescents (b) is significantly positive.*

**H4:**  *The indirect effect (a.b.) of digital game addiction (X) on academic motivation (Y) in adolescents, through RESE (M), is significantly negative.*

**H5:**  *The direct effect (c’) of digital game addiction (X) on academic motivation (Y) in adolescents is significantly negative.*

Hypotheses regarding the moderating role of PIU:

**H6:**  *The main effect of PIU (W) on academic motivation (Y) is significantly negative.*

**H7:**  *The effect of the interaction (X.W.) of digital game addiction (X) and PIU (W) on academic motivation in adolescents is significant.*

## 2. Methods

A correlation research methodology was employed to examine the mediating role of RESE and the moderating role of problematic internet usage on the relationship between digital game addiction and academic motivation. The correlation research model enables the researcher to test the relationship between variables. Because the correlation research model requires quantitative data, data were collected through self-report questionnaires that overlapped the theoretical frameworks of variables in the study. Data were analyzed through SPSS 22.0 and PROCESS macro software (version 4.3.1), which allows the researcher to test mediator and moderator analyses on the relationship.

### 2.1. Sample

The population in this study was high school students aged 15–18 years in Turkey. Because it is not possible to reach all high school students in Turkey, Muğla Province, located in Southwestern Turkey, was selected as the accessible population due to its convenience to the researchers. There were one hundred and two high schools in thirteen districts of Muğla Province at the time of data collection. Of these schools, thirty high schools (at least one high school from each district) were randomly selected for cluster sampling purposes. A total of 1156 high school students from different types of high schools (science, social science, vocational and Anatolian high schools) voluntarily participated in the study. Demographic characteristics of the sample were given in Table 1. As seen in Table 1, the majority of participants were female (68.1%) and ninth grade students (35.7%). Most of the participants were from Anatolian (73.1%) and urban high schools (80.4%). The participants reported that their parents were most likely to have a high school degree (35.6% for father and 30.7% for mother). Many participants reported using the internet for 3–4 h per day (35.5%) and mostly for the purpose of social media (58.8%).

### 2.2. Instruments

In this study, the following instruments were employed because they had been validated in a Turkish context and captured the target variables.

#### 2.2.1. Short Academic Motivation Scale (SAMS)

The SAMS, developed to measure students’ academic motivation, consists of 28 items and was created by Vallerand et al. (1989). Its Turkish adaptation was carried out by Yurt and Bozer (2015). The SAMS uses a 7-point Likert scale ranging from 1, “does not correspond at all”, to 7, “corresponds exactly”. Subsequently, Kotera et al. (2023) developed a short form of the SAMS, reducing the number of items to 14. In this study, the short form consisting of 14 items created by Kotera et al. (2023) was used. Higher scores on the scale indicate higher academic motivation, except for the amotivation subscale, which shows that the individual has no extrinsic or intrinsic motivation. Thus, two items in this subscale were reversed (Example items: “I go to school to attend a good high school, as my family wants”, “I go to school because it constantly encourages me to learn about topics that interest me”). In this study, the internal consistency score for the 14-item scale was found to be 0.88. For structural validity, confirmatory factor analysis (CFA) was performed. The results of CFA were (χ^2^ (77) = 427.56, CFI = 0.947, TLI = 0.927, RMSEA = 0.068, SRMR = 0.051). These results demonstrate that the SAMS is valid and reliable.

#### 2.2.2. Digital Game Addiction Scale (DGAS)

The DGAS, developed to measure adolescents’ digital game addiction, was created by Lemmens et al. (2009). The DGAS was adapted into Turkish by Yalçın-Irmak and Erdoğan (2015). It consists of seven items in a single dimension, using a five-point Likert-type scale. It is scored from 1, “Never”, to 5, “Always”. The scale does not include reverse-scored items, and higher scores indicate an increased risk of digital game addiction (Example items: “Have you ever thought about playing a computer game all day?”, “Did you feel bad when you couldn’t play a game?”). In this study, the internal consistency score for the 14-item scale was found to be 0.85. The CFA showed good fit indices (χ^2^ (14) = 45.21, CFI = 0.955, TLI = 0.928, RMSEA = 0.068). These results demonstrate that the DGAS is valid and reliable.

#### 2.2.3. Regulatory Emotional Self-Efficacy Scale (RESES)

The RESES, developed to measure individuals’ self-efficacy in regulating their emotions, was created by G. V. Caprara et al. (2008). The Turkish adaptation of the scale was carried out by Totan (2014). It is a 5-point Likert-type scale (1 = not well at all, 5 = very well) and consists of 12 items in three dimensions: (a) perceived self-efficacy in expressing positive affect (Example item: “How good are you at enjoying your achievements?”), (b) perceived self-efficacy in managing despondency/distress (Example item: “How good are you at avoiding a meltdown when you get angry?”), and (c) perceived self-efficacy in managing anger/irritation (Example item: “How good are you at quickly overcoming anger or frustration due to your mistakes?”). Higher scores on the scale indicate that individuals have higher abilities to manage their positive and/or negative emotions. In this study, the internal consistency score for the 12-item scale was found to be 0.83. The CFA showed good fit indices (χ^2^ (51) = 232.38, CFI = 0.924, TLI = 0.901, RMSEA = 0.062). These results demonstrate that the RESES is valid and reliable.

#### 2.2.4. Young’s Internet Addiction Scale Short Form (YIAT-SF)

The YIAT-SF is a short version of Young’s Internet Addiction Scale that Young (1998) developed by adapting the DSM-IV pathological gambling criteria into a 20-item scale, which was subsequently shortened to 12 items by Pawlikowski et al. (2013). Items in the YIAT-SF ask the individuals to rate “How often do you stay online longer than you planned?” and “How often do you try to hide how long you’ve been online?” on a five-point Likert scale (1 = rarely, 5 = always). The Turkish adaptation of the YIAT-SF was carried out by Kutlu et al. (2016). Targeting adolescents and university students, the scale aims to determine the level of internet addiction. Accordingly, in the long form of the scale with 20 items, those who score 80 and above are defined as “internet addicts”, those who score between 50–79 are “risky internet users”, and those who score 49 and below are “average internet users”. Although there is no specific cut-off point in the short form of the scale, higher scores indicate a higher level of internet addiction. Kutlu et al. (2016) found that the scale had a good fit for adolescents according to the confirmatory factor analysis results (χ^2^ (51) = 141.93, RMSEA = 0.080, GFI = 0.90, CFI = 0.90, and IFI = 0.90). The internal consistency reliability coefficient of the scale was calculated as 0.86. In this study, the internal consistency score for 12 items was found to be 0.85. The CFA had good fit indices (χ^2^ (54) = 224.74, CFI = 0.951, TLI = 0.936, RMSEA = 0.055). These results showed that the YIAT-SF was valid and reliable.

### 2.3. Data Collection and Analysis

After required permissions were granted, data were collected in the fall 2024 semester. In data collection, the following steps were taken. First, schools were visited to introduce the study. Once the principal of the schools agreed to participate in the study, data collection tools were administered to the potential participants under the supervision of their teachers. A class hour was given to the participants to fill out the data collection tools. In data analysis, the first, missing case and outlier were checked. A total of 14 cases were removed because their z-scores were around 4.00. Additionally, the normality of data was evaluated by checking kurtosis and skewness values. Because the kurtosis and skewness values of all variables were between −1.00 and 1.00, they were assumed to have a normal distribution (Tabachnick & Fidell, 2019). Then, confirmatory factor analyses with the structural model were performed to test the structural validity of the instruments. The literature suggests the acceptable fit indices as χ^2^/df < 2.5 or RMSEA < 0.08 or CFI > 0.90. Then, Cronbach alphas were computed to assess the internal consistency. The acceptable cut-off score was 0.70. CFAs were run in AMOS 18.0 software, whereas mediator and moderator analyses were done in SPSS 22.0 software by utilizing the PROCESS macro (Hayes, 2013). The study employed 95% bias-corrected bootstrap confidence intervals (CIs) derived from 5000 bootstrap resamples to assess the significance of conditional direct and indirect effects.

## 3. Results

Descriptive results for variables were given in Table 2. Mean values of the variables gave clues about high school students’ academic motivation, PIU, digital game addiction and emotional regulation self-efficacy. Mean value of academic motivation was found to be 3.54 (0.68). In the five-point scale, this mean value showed that Turkish high school students had a moderate level of academic motivation (1.00–2.33 for low, 2.34–3.66 for moderate, 3.67–5.00 for high). Mean value of PIU was 2.27 (0.66), which showed a low level of PIU. Mean value of digital game addiction was 1.82 (0.75) indicating that students had a low level of digital game addiction. Mean value of RESE was 3.11 (0.75), showing that students had a low level of positive and negative emotional regulation self-efficacy. Pearson correlation coefficients in Table 2 showed that academic motivation was positively and significantly correlated with RESE (*r* = 0.18, *p* < 0.01) but negatively with digital game addiction (*r* = −0.21, *p* < 0.01) and PIU (*r* = −0.19, *p* < 0.01). PIU was positively correlated to digital game addiction (*r* = 0.48 *p* < 0.01); yet negatively with RESE (*r* = −0.29, *p* < 0.01). Lastly, digital game addiction was negatively correlated to RESE (*r* = −0.13, *p* < 0.01).

### Results of Mediation and Moderator Analysis

**Figure 2:**
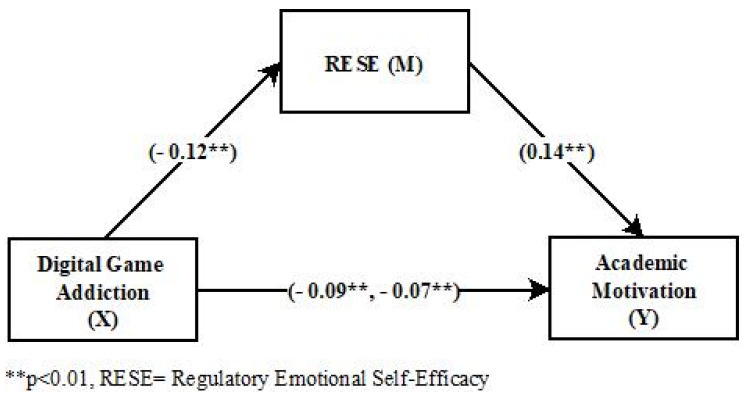
The total, direct and indirect path coefficients for the mediating role of RESE in the effect of digital game addiction on academic motivation.

Standardized regression coefficients and confidence intervals related to the examination mediating role of RESE on the interplay between digital game addiction and academic motivation were given in Table 3 and Figure 2. As seen in Figure 2, the total effect of digital game addiction on academic motivation (c) is negatively significant (*β* = −0.09, 95% CI [−0.13, −0.04], *p* < 0.05). This result showed that H1 was supported. On the other hand, the path coefficients related to the direct effects between the variables showed that the direct effect of digital game addiction on RESE was negatively significant (*β* = −0.12, 95% CI [−0.16, −0.07], *p* < 0.05). The direct effect of RESE on academic motivation (b) (*β* = 0.14, 95% CI [0.09, 0.20], *p* < 0.05) and the direct effect of digital game addiction on academic motivation (c’) (*β* = −0.07, 95% CI [−0.12, −0.03], *p* < 0.05) were statistically significant, supporting H3 and H5. The indirect effect (ab) of digital game addiction on academic motivation through RESE was found to be negatively significant (*β* = −0.02, 95% CI [−0.03, −0.007], *p* < 0.05), indicating that H4 was supported. To sum up, these results showed that the direct effect of digital game addiction on academic motivation and the indirect effect through RESE were significant.

In Table 4, results regarding the moderator role of PIU on the effect of digital game addiction on academic motivation were given. The model established regarding the prediction of academic motivation by digital game addiction, PIU and interaction variables was significant (*F* (2,1151) = 5.744, *p* = 0.000]. These three variables together predicted academic motivation and explained 12% of its variance. PIU negatively and significantly predicted academic motivation, which supported H6. In addition, the interaction term (X × the amount of change in the explained variance by adding W) statistically significantly predicted academic motivation [*R*^2^_(change)_ = 0.006, *F* (1,1151) = 7.629, *p* = 0.010), indicating that H7 was supported. Accordingly, the significant coefficient of the interaction term predicting academic motivation and the confidence intervals not containing zero and being in the same direction as the standardized beta coefficient indicated that PIU had a moderating effect on the effect of digital game addiction on academic motivation.

Findings regarding the significance and confidence intervals of the effect of digital game addiction on academic motivation for different levels of PIU (low −1 sd, average and high +1 sd) were given in Table 5. As seen in Table 5, the effect of digital game addiction on academic motivation varied at different levels of PIU. When the level of PIU was low and medium, the negative effect of digital game addiction on academic motivation was statistically significant. However, when PIU was high, the negative effect of digital game addiction on academic motivation became statistically insignificant. Accordingly, it can be said that a high level of PIU plays a moderating role in the effect of digital game addiction on academic motivation. In other words, when the level of PIU is high, digital game addiction negatively affects academic motivation. It can be said that the basis of the problem lies in whether students play digital games or not, but also in their high level of PIU.

**Figure 3:**
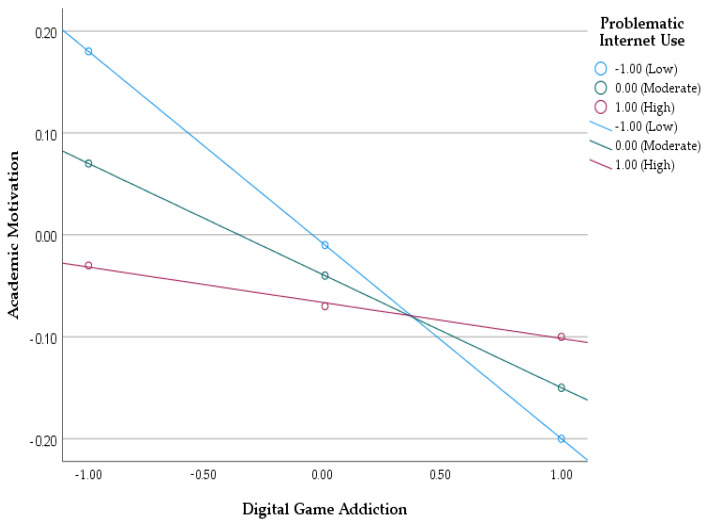
The moderating role of digital game addiction on academic motivation at different levels of PIU.

Figure 3 showed the moderating role of PIU in the effect of digital game addiction on academic motivation at low, medium and high levels. When the effect of digital game addiction on academic motivation is examined at different levels of PIU, it can be said that the effect of digital game addiction on academic motivation is significant when the level of PIU is low and medium; however, this effect is insignificant when the level of PIU is high. In short, it can be said that when PIU is high, the effect of digital game addiction on academic motivation becomes insignificant and it assumes a full mediating role in the relationship.

## 4. Discussions

The purpose of this study was to examine the mediating role of RESE and the moderating role of PIU in the relationship between digital game addiction and academic motivation among Turkish adolescents. Self-report questionnaires were employed to measure these variables, and statistical analyses were conducted to address the research questions.

The descriptive results provided insights into Turkish adolescents’ levels of PIU, academic motivation, RESE, and digital game addiction. The mean value for academic motivation indicated that Turkish adolescents had a moderate level of motivation on a five-point scale. This finding aligns with the results of Ulum and Alkış-Küçükaydın (2024), who reported a similar level of academic motivation (M = 4.46 on a seven-point scale) in their study of Turkish adolescents. Likewise, the participants in this study demonstrated a moderate level of emotional regulation, consistent with findings from Demirtaş (2020) that Turkish high school students generally have moderate levels of RESE.

However, the mean value for PIU (as measured by the Young’s Internet Addiction Test—Short Form) suggested that Turkish adolescents exhibited a low level of problematic internet use, which was also consistent with previous studies (e.g., Çelebioğlu et al., 2020). Similarly, the mean value for digital game addiction was low, in line with previous research in Turkey (e.g., Sağlam & Turan, 2024). These descriptive results suggest that the sample and measurement tools used in this study align well with the existing literature, supporting their external validity.

Within the scope of this study, seven hypotheses were tested, and all were accepted. The results confirmed a negative causal relationship between digital game addiction and academic motivation, as hypothesized (Hypothesis 1 was accepted). In other words, adolescents with higher levels of digital game addiction were more likely to exhibit lower academic motivation. This finding is consistent with Sahin et al. (2016), who reported that game addiction negatively impacted students’ academic performance. The result is plausible, as gaming can distract students from their studies and diminish their interest in academic pursuits.

The study also found that RESE had a direct positive effect on academic motivation (Hypothesis 3 was accepted). Adolescents with higher competence in regulating their emotions tended to have higher academic motivation. This result is supported by the existing literature. For instance, Gumora and Arsenio (2002) highlighted the significant role of emotional factors in students’ academic success, suggesting that effective emotion regulation enhances academic motivation (Zhang et al., 2022). Thus, this study underscores the importance of strategies aimed at improving students’ emotion regulation skills to boost academic performance.

One key finding was that RESE played a partial mediating role in the relationship between digital game addiction and academic motivation (Hypothesis 2 was accepted). Although the indirect effect of digital game addiction on academic motivation remained statistically significant (Hypothesis 4 was accepted), the direct effect of digital game addiction decreased from −0.09 to −0.02 when RESE was included as a mediator (Hypothesis 5 was accepted). This suggests that confidence in regulating emotions can help mitigate the negative impact of digital game addiction on academic motivation. Previous research also supports the connection between RESE and a lower likelihood of digital game addiction and social media addiction (Giordano et al., 2023).

Additionally, the study found that PIU moderated the relationship between digital game addiction and academic motivation (Hypothesis 6 was accepted). Specifically, when PIU levels were “low” or “moderate”, the negative effect of digital game addiction on academic motivation was reduced but still significant. However, when PIU levels were “high”, the negative effect became statistically insignificant, indicating a buffering effect (Hypothesis 7 was accepted). This finding suggests that high levels of PIU may serve as a maladaptive coping strategy, potentially reducing the negative consequences of digital game addiction on academic motivation, as proposed by Gioia et al. (2021).

Similarly, Fontana et al. (2023) demonstrated a buffering role of PIU in the relationship between adolescent identity and internalized problems, as well as between aggression regulation and internalized problems, further supporting the role of PIU as a coping mechanism. However, high levels of PIU should still be considered a risk factor, as they may have negative consequences in other areas of adolescents’ lives.

In summary, this study highlights the need to consider PIU when examining the effect of digital game addiction on academic motivation. Studies such as Donoso et al. (2021) support the notion that high levels of PIU can have a moderating effect, consistent with the findings of this study. Moreover, Wang et al. (2024) found that PIU played a full mediating role between entertainment-seeking behaviors and self-harm, and a partial mediating role between reward-seeking behaviors and self-harm. Zhao et al. (2024) also reported that PIU moderated the relationship between digital nativeness and general functioning in adolescents, demonstrating how high levels of PIU can affect various developmental outcomes.

## 5. Conclusions

This study provided evidence that digital game addiction is a negative predictor of academic motivation among Turkish adolescents. Furthermore, the findings suggest that emotional regulation self-efficacy partially mediates the adverse impact of digital game addiction on academic motivation. Additionally, the study revealed that the negative effect of digital game addiction on academic motivation is moderated by problematic internet use (PIU), with the effect becoming significant only at high levels of PIU. This conclusion highlights the complex interplay between digital game addictions, academic motivation, RESE, and PIU, emphasizing the need for targeted interventions to mitigate the negative consequences on students’ academic motivation.

## 6. Recommendations

The results of this study suggested that enhancing adolescents’ RESE is crucial to lessening the negative impacts of digital game addiction on academic motivation. In this context, school-based psychosocial programs or emotional intelligence instruction may be helpful. For instance, students could be encouraged to engage in mindfulness content that emphasizes self-regulation and emotional well-being or to engage in physical activities and cognitive restructuring. Another measure could be implementing weekly check-in sessions where students can discuss their gaming habits and academic process with counselors.

It can be suggested that the severity of the issue is highlighted by the finding that a high prevalence of PIU reduces the relationship between digital game addiction and academic motivation. As a result, it is critical that parents and teachers keep an eye on their teenagers’ internet usage and impose limits as necessary. In an effort to help students become more aware of addictive behaviors and give them the skills they need to regulate their use of digital media, schools can implement instructional programs that increase awareness of digital game addiction and PIU. For this, students can be introduced to apps that promote digital well-being, allowing them to monitor their gaming time and set limits. Furthermore, individual therapy may be a useful strategy for resolving the negative consequences of these issues on their lives.

## 7. Limitations

There are several issues that should be taken into consideration before generalization of the study across other groups. First, because gender is an important determinant in digital game addiction (boys tend to be more game-addicted than girls; André et al., 2022; Türen & Bağçeli Kahraman, 2024), unequal distribution of gender in the sample of this study should be taken into consideration. Moreover, the sample size is one of the important factors that make a regression test statistically significant. Thus, the statistical significance in this study could be due to the large sample size of the study.

## Figures and Tables

**Table 1:** Demographic characteristics of the sample.

		n	%
Gender	Female	787	68.1
	Male	369	31.9
Grade	9th	413	35.7
	10th	23	19.3
	11th	298	25.8
	12th	222	19.2
School type	Anatolian	845	73.1
	Science/Social Science	83	67.1
	Vocational/Art	224	19.5
Father/mother education level	Primary school	293/373	25.3/32.3
	Middle school	236/244	20.4/21.1
	High school	412/355	35.6/30.7
	Undergraduate	183/164	15.8/14.2
	Graduate	32/20	2.8/1.7
School location	Rural	226	19.6
	Urban	930	80.4
Internet use frequency	1–2 h a week	36	3.1
	3–4 h a week	53	4.6
	1–2 h in a day	407	35.2
	3–4 h in a day	410	35.5
	More than 4 h a day	250	21.6
Purpose of internet use	Social media	680	58.8
	Education/Scientific research	167	14.4
	Communication	91	7.9
	Game	178	15.4
	News/article reading	22	1.9
	Personal (banking, shopping)	18	1.6

**Table 2:** Descriptive statistics regarding the variables and correlation coefficients (r).

Variables	1	2	3	4	5	6
1—Gender	1					
2—IUF	0.029	1				
3—AM	−0.108 **	−0.033	1			
4—PIU	−0.040	−0.055	−0.185 **	1		
5—DGA	0.359 **	−0.016	−0.206 **	0.477 **	1	
6—RESE	0.207 **	0.046	0.181 **	−0.292 **	−0.126 *	1
$\bar{x}$	--	3.68	3.54	2.27	1.82	3.11
Sd.	--	0.96	0.68	0.65	0.75	0.70
S	--	−4.84	−0.45	0.75	0.93	0.17
K	--	0.153	0.13	0.15	0.33	0.08
α	--	--	0.88	0.85	0.85	0.83

* *p* < 0.05,** *p* < 0.01, N = 1134, S = Skewness, K = Kurtosis, α = Cronbach alpha, IUF = Internet Use Frequency, AM = Academic Motivation, DGA = Digital Game Addiction, PIU = Problematic Internet Use, RESE = Regulatory Emotional Self-Efficacy.

**Table 3:** Total, direct, indirect effects and confidence intervals between research variables (in parentheses).

Paths	Total Effect	Direct Effect	Indirect Effect	Decision
1—DGA → AM	−0.09 ** (−0.13, −0.04)			Partial Mediation
2—DGA → RESE		−0.12 ** (−0.16, −0.07)		
3—RESE → AM		0.14 ** (0.09, 0.20)		
4—DGA → RESE → AM			−0.02 * (−0.03, −0.007)	

AM: Academic motivation, DGA: Digital game addiction, RESE, ** *p* < 0.00, * *p* < 0.05.

**Table 4:** Regression results relating to moderating role of PIU on effect of DGA to AM.

Model	B	SE	*t*	*p*	LLC	ULCI
Constant	52.372	2.2796	22.974	<0.001	47.899	56.844
DGA (X)	−0.60	0.1837	−3.270	<0.001	−0.961	−0.072
PIU (W)	−0.23	0.0796	−2.872	0.004	−0.385	−0.240
X × W	−0.16	0.005	2.276	0.005	0.004	0.027
*R* = 0.12, *R*^2^ = 0.2, F (3,1150) = 5.744, *p* < 0.001						
X × W, *R*^2^_(change)_ = 0.067, *F* (1, 1150) = 7.629, *p* = 0.005, Dependent variable: Academic motivation						

**Table 5:** The effect of digital game addiction on academic motivation at different levels of PIU (low, medium, and high).

PIU	B	SE	*t*	*p*	LLC	ULCI
Low (−1 ss)	−0.30	0.08	−3508	<0.001	−0.469	−0.132
Average	−0.18	0.06	−3050	0.002	−0.287	−0.062
High (+1 ss)	−0.05	−0.06	−0.843	0.399	−0.163	−0.065

## Data Availability

Data are contained within the article.
